# Supplementary material for: Synergistic Effects of Venetoclax and Daratumumab on Antibody-Dependent Cell-Mediated Natural Killer Cytotoxicity in Multiple Myeloma
Source: Int J Mol Sci. 2021 Oct 5;22(19):10761. doi: 10.3390/ijms221910761 (PMC8509545; doi:10.3390/ijms221910761)
Supplement: Supplementary file 1 [file ijms-22-10761-s001.zip › ijms-1267187-supplementary.pdf]

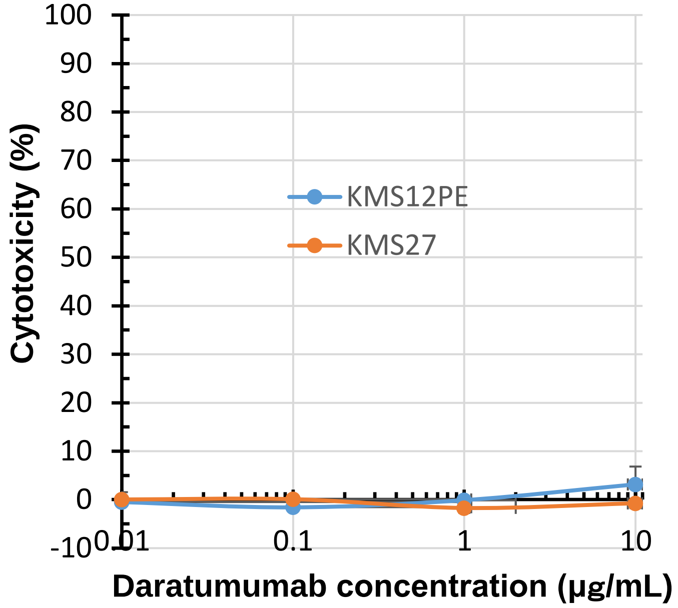

Supplementary Figure S1. Cytotoxicty of daratumumab induced complement dependent cytotoxicity (CDC) to MM cell lines. The MM cell lines KMS12PE and KMS27 were used as target cells for CDC activity assessment. KMS12PE or KM5 were incubated with serial concentrations of daratumumab for 3h in RPMI1640 containing 20% human serum as a source of complement. These cells were stained with 10 μg/mL propidium iodide after fixation with 70% EtOH for 20 min at -20℃. The stained cells were analyzed by flow cytometry. Cytotoxicity was calculated as follows: cytotoxicity (%) = [(the measured value at each daratumumab concentration) - (the measured value when no daratumumab was used)] / [100 - (the measured value when no daratumumab was used) × 100] and is shown as the mean of three different assays.
